# Patch Augmentation in Arthroscopic Rotator Cuff Surgery—Review of Current Evidence and Newest Trends

**DOI:** 10.3390/jcm13175066

**Published:** 2024-08-27

**Authors:** Maximilian Russo, Gert Karl Dirkx, Claudio Rosso

**Affiliations:** 1 Departement of Orthopedics and Traumatology, Spitäler fmi AG, 3800 Interlaken, Switzerland; 2 ARTHRO Medics, 4054 Basel, Switzerland; 3 Regionaal Ziekenhuis Tienen, 3300 Tienen, Belgium; 4 Orthopedics and Trauma Surgery Clinic, University of Basel, 4001 Basel, Switzerland

**Keywords:** shoulder, rotator cuff repair, rotator cuff patch, review of literature

## Abstract

**Background:** Rotator cuff tears are a common and debilitating condition requiring surgical intervention. Arthroscopic rotator cuff repair is essential for restoring shoulder function and alleviating pain. Tear classification by size and tendon retraction, along with the grade for fatty infiltration, influence postoperative outcomes, with large tears and higher fatty infiltration grades linked to higher retear rates. Managing complex tears is challenging, with failure rates ranging from 20 to 94%. Patch augmentation has emerged as a promising strategy, using biological or synthetic materials to reinforce tendon repairs, enhancing structural integrity and reducing retear risk. **Methods:** A review of the recent literature from January 2018 to March 2024 was conducted using PubMed/MEDLINE, Embase, and Web of Science. Keywords included “rotator cuff tear”, “rotator cuff augmentation”, “rotator cuff patch”, “tendon augmentation”, “massive rotator cuff tear”, “patch augmentation”, and “grafts”. Relevant articles were selected based on their abstracts for a comprehensive review. **Results:** Initial methods used autograft tissues, but advances in biomaterials have led to standardized, biocompatible synthetic patches. Studies show reduced retear rates with patch augmentation, ranging from 17 to 45%. **Conclusions:** Patch augmentation reduces the retear rates and improves tendon repair, but complications like immune responses and infections persist. Cost-effectiveness analyses indicate that while initial costs are higher, long-term savings from reduced rehabilitation, revision surgeries, and increased productivity can make patch augmentation economically beneficial.

## 1. Introduction

### 1.1. Rotator Cuff Tears

Rotator cuff tears represent a common and debilitating musculoskeletal condition characterized by the disruption of the tendons surrounding the shoulder joint and are strongly variable in size. Arthroscopic rotator cuff repair is a key surgical treatment option for rotator cuff tears, allowing patients to have improved shoulder function and reduced pain with minimally invasive techniques [1].

Rotator cuff tears can be classified according to the tear size into small (<1 cm), medium (1–3 cm), large (3–5 cm), and massive (>5 cm) tears, according to DeOrio and Cofield [2].

Retraction of the tendon can be classified according to Patte. Together with the tendon repair quality, retraction type Patte 3 is associated with a higher chance of postoperative retear [3]. One other important aspect of assessing the success of rotator cuff surgery is the classification of Goutallier. This classification system, developed in the 1980s, categorizes the severity of fatty infiltration within the rotator cuff muscles in 5 grades, from normal to grade 4, meaning more fat than muscle. A grade 3 or 4 is associated with increased failure of repair with rates of 76% and 100%, respectively [4]. In traumatic supraspinatus tendon tears, there is a strong positive correlation between the time of injury, fatty infiltration and the level of retraction, as described by Ilyas et al. [5]

The management of these complex tears, compromised tissue quality, and challenges associated with tendon healing present an ongoing challenge with failure of structural healing rates for large to massive tears of 20–94% [3,6,7,8].

In recent years, the concept of patch augmentation has emerged as a promising adjunctive strategy to address these challenges and optimize outcomes in arthroscopic rotator cuff surgery [9,10]. It involves the application of biological or synthetic materials to reinforce the native tendon repair, augment tissue regeneration, and enhance biomechanical strength. By hoping to provide structural support and promote healing at the repair site, patch augmentation holds the potential to improve the integrity and durability of rotator cuff repairs, thereby reducing the risk of retears and enhancing functional recovery [11].

In cases of irreparable posterosuperior massive rotator cuff tears, a patch-based approach known as superior capsule reconstruction is also employed. This technique involves securing a patch between the upper glenoid rim and the greater tuberosity. Its goal is to mitigate humeral head subluxation, thus restoring joint stability, but this technique is out of the scope of this review [12].

The earliest reports of applications of patch augmentation of the rotator cuff date back to the late 1980s, with increased interest from the early 2000s onwards. Multiple techniques, open or arthroscopic, and different kinds of patches have arisen since [13,14].

Advancements in biomaterials science and tissue engineering soon led to the development of synthetic patches specifically designed for the purpose of rotator cuff augmentation. These patches should offer advantages such as standardized dimensions, biocompatibility, and the absence of donor site morbidity, thus revolutionizing the field of rotator cuff surgery. Retear rates with patch augmentations are reduced and are currently described from 17–45% [15,16,17].

### 1.2. Augmentation vs. Interposition

Patch augmentation and interposition are two surgical techniques used in the management of rotator cuff tears, each with its own distinct approach and purpose. Patch augmentation involves using a graft or a patch to reinforce and augment the repair of a torn rotator cuff tendon. The patch is typically fixed on top of the repaired tendon with sutures or anchors to provide additional strength and support to the healing tissue. The primary goal of patch augmentation is to enhance the structural integrity of the repair and improve the chances of successful healing, particularly in cases of large tears. Patch augmentation is indicated in patients displaying additional risk factors that could potentially lead to failure after a non-augmented rotator cuff repair, including advanced age, considerable retraction, muscular atrophy and fatty infiltration, and reduced bone mineral density. Symptomatic primary or revision rotator cuffs can tear, especially after a persistent shoulder pain situation and functional deficits after conservative treatment. Common patch materials include synthetic grafts, allografts, or xenografts [18].

Unlike patch augmentation, where the patch is placed on top of the tendon, interposition involves inserting the graft or spacer into the space between the tendon and the bone. The primary purpose of interposition is to provide a barrier, reducing friction and pressure on the healing tissue. Interposition can be particularly beneficial in cases where there is significant tendon retraction or when the torn tendon cannot be directly repaired to the bone, so as large tears or irreparable cuff tears. Common interposition materials include autografts, allografts, or synthetic spacers [18].

## 2. Materials and Methods

**Figure 1:**
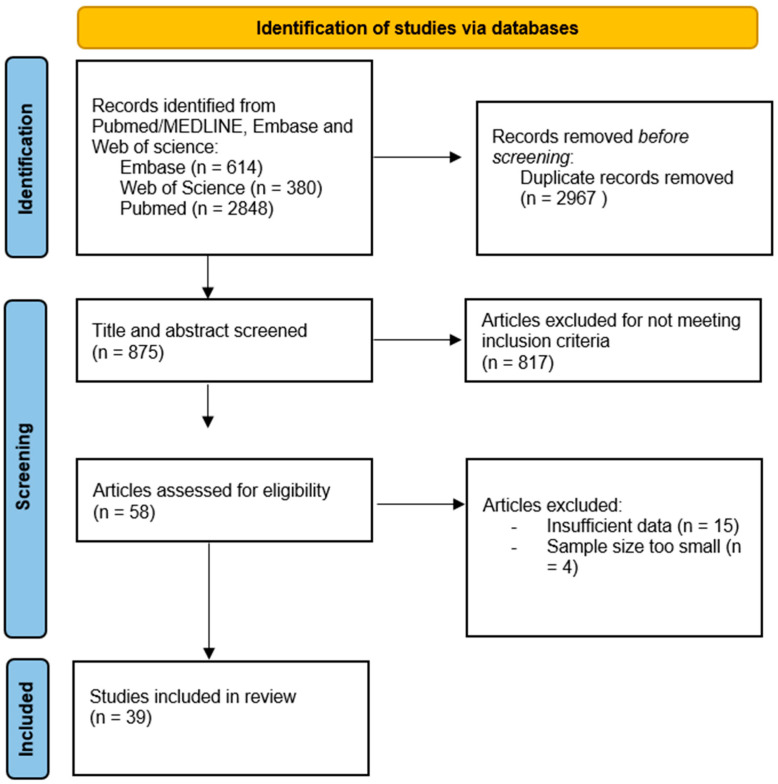
PRISMA flowchart.

Relevant articles focusing on rotator cuff augmentation were extracted by all the authors by using a thorough database (PubMed/MEDLINE, Embase and Web of Science) using the PRISMA guidelines for identifying and evaluating relevant studies (Figure 1). The search conducted from March to June 2024 employed a combination of the following keywords: “rotator cuff tear” and/ or “rotator cuff augmentation” and/ or “rotator cuff patch” and/ or “tendon augmentation” and/ or “irreparable rotator cuff tear” and/ or “patch augmentation” and/ or “grafts” and hereby focusing on articles of the last 5 years (January 2018–March 2024). A total of 3842 articles were retrieved. Two independent researchers, G.D. and M.R., assessed the eligibility of each article by thoroughly reviewing their abstracts (see Table 1). If there was any disagreement, the third researcher, C.R., was brought in to achieve consensus. Before abstract screening, articles were removed due to duplicates or not relevant to rotator cuff repair. In total, 875 abstracts were screened, and 817 articles were excluded due to missing information about rotator cuff augmentation, single surgeon technique propositions or techniques other than patch augmentation. In total, 58 articles were assessed for eligibility. Hereby, another 21 were excluded due to insufficient data or too small sample size. In total, 39 are included, from which a comprehensive review is provided.

## 3. Results

### 3.1. Different Graft Types

#### 3.1.1. Xenografts

Xenografts from animal tissue are widely explored and used in treating skin loss as substitutes and are mostly of porcine or bovine origin. Initially, for rotator cuff treatment, the studies were with dermal grafts [19,20] and showed fast incorporation and conversion, but rejections were reported [21]. This is mainly due to residual foreign DNA or galactose-alfa-1,3-galactose, more specifically in porcine-derived products. However, there was a wide variability between different scaffolds in containing original DNA remnants as some contained. These studies suggest only a limited mechanical role in augmentation [22,23,24]. In 2017, a level IV case study was published by Neumann et al., in which they evaluated clinical and morphological outcomes of a porcine dermal matrix xenograft Conexa (Tornier, Edina, MN, USA) used for interposition in arthroscopic rotator cuff repair in 61 patients. At an average follow-up duration of 50.3 months, the findings unveiled significant improvements in pain relief, range of motion, and muscle strength among the patients. Moreover, postoperative ultrasound assessments indicated a remarkable 91.8% preservation of repair integrity at the final follow-up. Furthermore, no infections, signs of inflammatory response, tissue rejection, or significant adverse events were observed among any participants in this study [25]. Flury et al. examined porcine xenografts in cuff repair, finding that after 24 months, recurrent SSP tendon defects occurred in four control group patients (*n* = 20) and nine patch group patients (*n* = 20). However, the difference was not statistically significant. Most defects (85%) were medial cuff failures, predominantly in the patch group. Pain decreased similarly in both groups post-surgery. No significant group differences are observed in other outcomes, and recurrent defects did not notably affect function. Local complications, including recurrent defects, were comparable between groups. The study also noted significant tissue inflammation in 12 of 20 patients after porcine xenograft use [15].

#### 3.1.2. Allograft and Autograft

**Figure 2:**
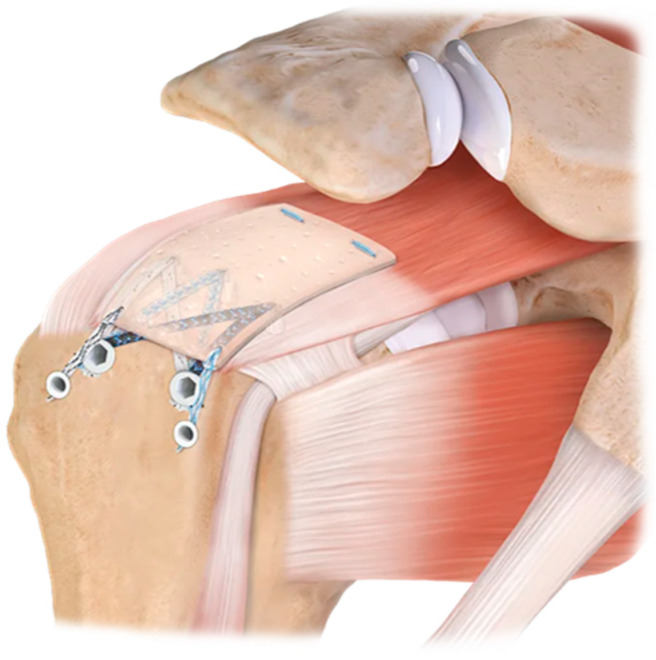
Patch augmentation with a patch allograft. Image courtesy of ARTHREX.

Human allograft patches originate from dermal tissue or fascia lata. Compared to xenografts, human skin has higher loads-to-failure compared with porcine and bovine skin and this is to small intestine mucosa (see Figure 2) [16]. The grafts do differ in failure modes in which each graft had a specific tendency of failure. Although all scaffolds failed through suture pullout, there were three clear patterns. First of all, a pullout through the isthmus is described in which the sutures pull through the stitch interval. Secondly, there can be a side pullout, where they pull out of both sides of the graft, and thirdly, when the sutures are pulled directly through the graft, it is described as an end pullout. Barber et al. described only one graft breakage [26]. Despite the fact that in vitro studies are promising, in vivo performance can still be poor. Pullout strength of the implants is important to guarantee immediate mechanical benefit. Decellularized human skin, while displaying comparable mechanical failure load and strength to cellularized skin, showcases a significantly higher stiffness in its matrix [24]. This distinguishes it from natural tendons, which have a stiffness of approximately three orders of magnitude greater. Consequently, the limited mechanical role of decellularized skin in tendon augmentation suggests a similarity to xenografts [27].

Kim et al. highlighted the use of the ADM patch (Bellacell; HansBiomed, Seoul, Republic of Korea) as augmentation in incomplete rotator cuff repair using a “hybrid technique”, where the anterior border of the greater tubercle of the humerus remains exposed due to bad tissue quality. By utilizing patch augmentation to cover the incomplete repair and increasing the thickness of the repaired tendon, the incidence of retear was lower compared with the group undergoing rotator cuff repair alone, considering the procedure’s increased complexity and time requirements [10]. Subsequent a prospective randomized multicenter study also showed a significantly higher retear rate in the control group (*n* = 22) other than the patients treated with dermal allograft augmentation (*n* = 20) after rotator cuff repair measured with magnetic resonance imaging at a mean follow-up of 2 years, with no graft-related adverse events [28].

Mori et al. demonstrated in a case series involving 45 patients with massive posterosuperior rotator cuff tears and varying degrees of fatty degeneration in the infraspinatus and supraspinatus muscles that the arthroscopic patch interposition procedure using a fascia lata autograft significantly improved ASES and Constant scores at the final follow-up. However, patients with preoperative high-grade fatty degeneration of the infraspinatus and supraspinatus did not achieve favorable results compared with other treatment options [29].

**Figure 3:**
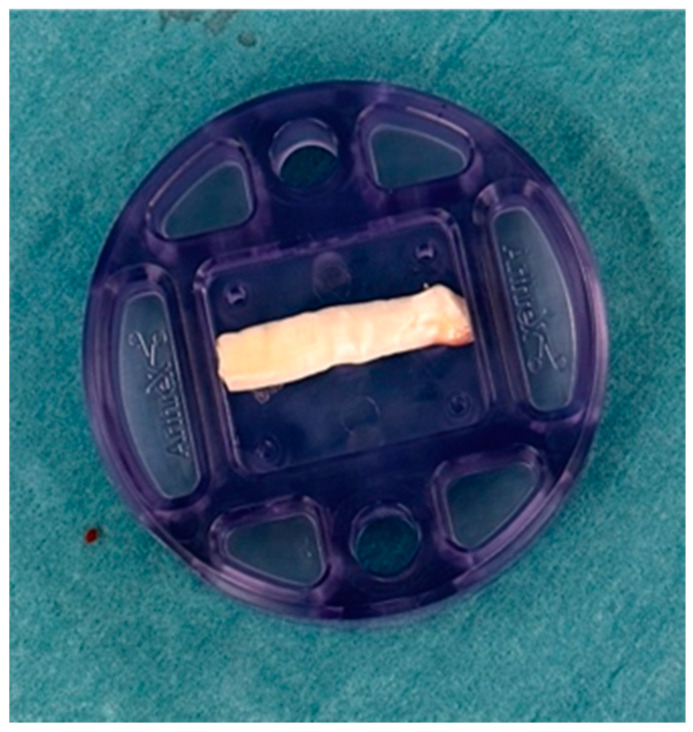
The long head of bicep tendon is cut to appropriate size.

**Figure 4:**
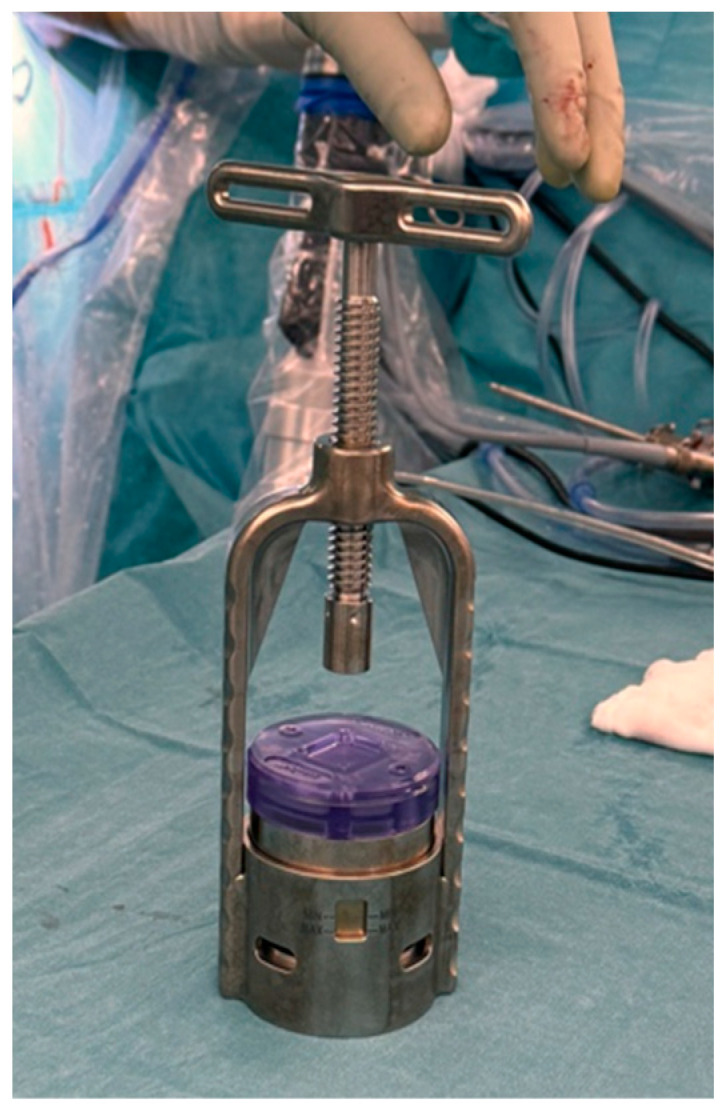
The tendon press is manually tightened and left in this position for 3 minutes.

**Figure 5:**
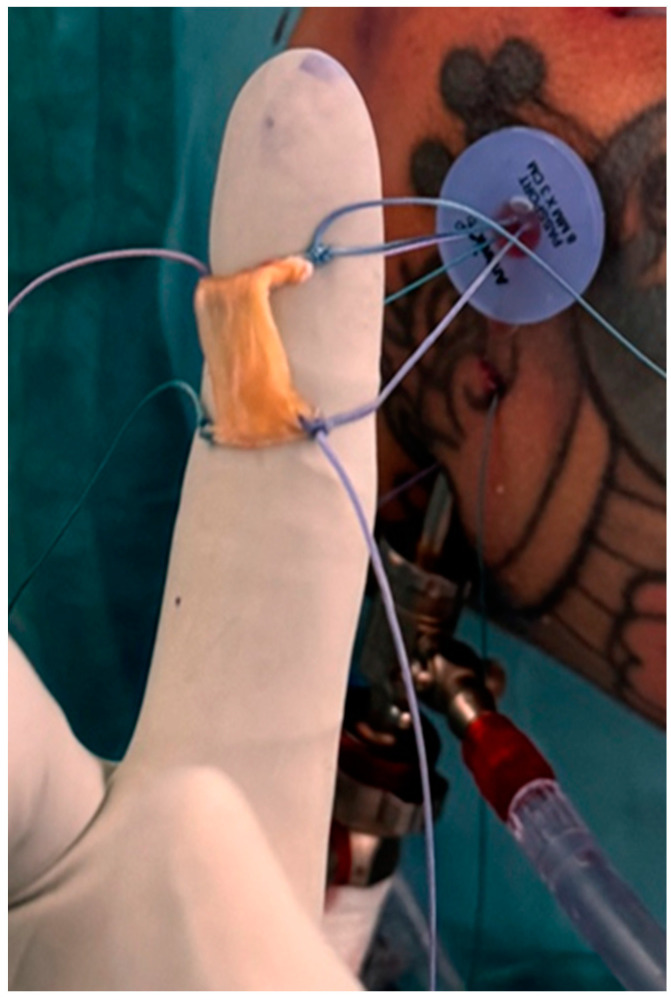
The patch is reinforced extracorporeally with four sutures, which are pulled in using the provided corner sutures.

A readily available autograft is the long head of the biceps tendon (LHBT). If no previous biceps surgery has been performed, the proximal part can be used as a graft. This comes without the increased initial cost compared with the commercially available grafts and without any donor site morbidity. Ideally, the non-tendinopathic (most distal) part is used, and the rounded tendon is cut in length to the appropriate size and then flattened out into a rectangular structure by a press (see Figure 3 and Figure 4). The final graft size is to be expected at +/−25 mm × 15 mm [30] (see Figure 5). If performing a suprapectoral biceps tenodesis, the harvested portion length might be too little, although Hohmann proposed to mesh it like a split thickness autograft. This can enlarge the graft and make it more porous [31]. Colbath et al. showed that this graft is biologically active, meaning that tenocytes in this graft produce adequate signals to differentiate adipose-derived mesenchymal stem cells into immature tenocytes [32].

Very few comparative studies between the LHBT graft and commercially available grafts exist. In a retrospective study of Sung with 32 patients with irreparable cuff tears, a graft was used for bridging. A total of 24 patients received the LHBT graft, whilst 8 received an allogenic dermal allograft. Healing failures at 1 year postoperatively were 54.2% and 75% in the LHBT group and the allograft group, respectively, but not significantly different (*p* = 0.4) [33]. Park et al. retrospectively reviewed 77 patients with incomplete repair of large or massive rotator cuff tears where 30 of them received a LHBT augmentation. They found no significant difference in retear rates or clinical outcomes [34]. As a bridging structure for irreparable cuffs, this does not seem to work. As an augmentation, theoretical evidence can support this graft, although comparisons with commercially available augmentations are rare to none.

#### 3.1.3. Synthetic Polymers

Innovative manufacturing methods have facilitated the replication of desired tissue characteristics by crafting complex nano-scaffolds and biologically enhanced grafts. Different materials (such as polypropylene, carbon, PTFE, and silicon) and polymers (such as nylon) have been used [11,35,36]. However, studies showed various results with synthetic grafts. In 2018, a retrospective study was published that evaluated the long-term clinical outcomes of a synthetic polyester graft Dacron (DuPont, Wilmington, DE, USA) used for interposition with screw fixation in arthroscopic rotator cuff repair. The study found that the clinical and radiological outcomes at almost 20-year follow-ups were poor, indicating that such grafts were unable to prevent further cuff tear arthropathy or maintain cuff integrity in the long term. Additionally, the mean Constant-Murley (CM) score after at least 17 years was 46, doubting the long-term effectiveness of synthetic interposition grafts [37].

A newer prospective cohort study conducted by Smolen et al. in 2019 demonstrated favorable clinical outcomes after rotator cuff reconstructions augmented with a synthetic polyester patch (Pitch-Patch, Xiros Inc., Mansfield, MA, USA), revealing a substantial improvement in Constant-Murley scores and subjective shoulder value at follow-up. All 50 participants underwent arthroscopic rotator cuff reconstruction, with pre- and post-operative assessments of tendon integrity using magnetic resonance imaging (MRI) and computed tomography (CT), supplemented by ultrasound examination. Furthermore, a retear rate of 14% was reported in a mean follow-up of 8 months. The study groups highlighted a significant correlation between retear rates and preoperative retraction grade 3 to Patte [38]. However, there was no correlation observed with fatty infiltration.

Mechanical properties: When it comes to suture pull-through, synthetic patches like SportMesh (Biomet, Warsaw, IN, USA) exhibit significantly higher stiffness and stability compared with allografts, autografts, and xenografts (approximately 500 J/cm^3^ vs. 150 J/cm^3^). On the other hand, synthetic mesh demonstrates a significantly lower tensile (Young’s) modulus (approximately 14 MPa at 2–5% strain) compared with xenografts as ZCR (Zimmer, Warsaw, IN, USA) (approximately 68 MPa at 29–37% strain) and allografts as Graft Jacket (Wright Medical, Memphis, TN, USA) (approximately 48 MPa at 27–36% strain). The extracellular matrices derived from small intestine submucosa (ZCR) had higher moduli than the dermis-derived extracellular matrices (Graft Jacket). However, at strains of 2% relevant to physiological conditions, SportMesh and Graft Jacket exhibited significantly higher moduli compared with ZCR (7 MPa vs. 13 MPa and 19 MPa) [36].

#### 3.1.4. The Future of Patch Augmentation

**Figure 6:**
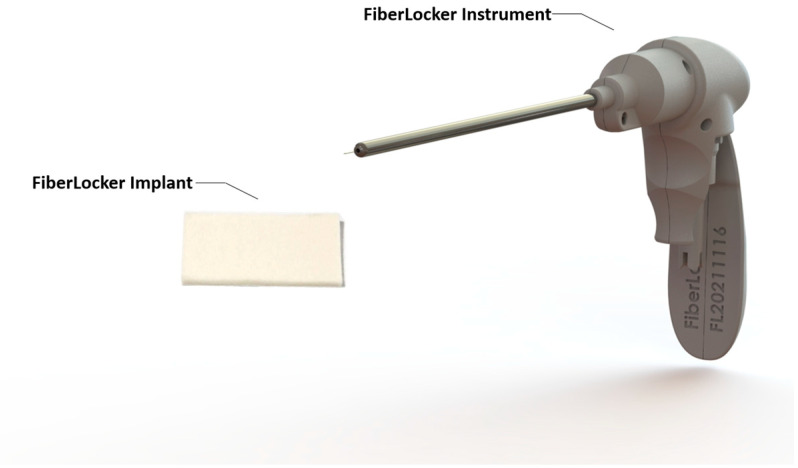
The FiberLocker System is composed of two essential elements: firstly, the FiberLocker Implant, constructed from polyester (polyethylene terephthalate) fibers to bolster rotator cuff repairs, and secondly, the FiberLocker Instrument.

**Figure 7:**
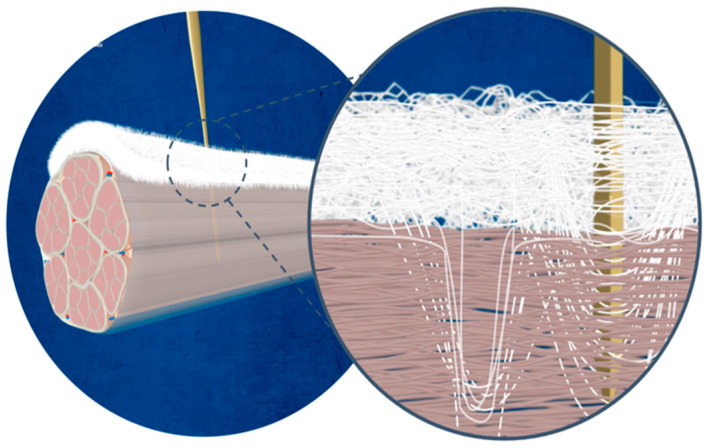
The FiberLocker Instrument, a surgical micro-stitching device, is meticulously designed for precisely securing the FiberLocker Implant within soft tissue. Courtesy of ZuriMED Technologies AG (Zürich, Switzerland).

An important downside of patch implantation and its rotator cuff augmentation is the fixation of the patch itself to the repaired tendon. Currently, patches are fixed with 6–10 fixation points using either special devices adapted from meniscal repair systems or bypassing sutures through the tendon and graft, respectively. An advancement is currently being developed; as can be seen in Figure 6 and Figure 7, as a main function, a needle is passed through a synthetic patch and consecutively through the tendon like a sewing machine. In this way, the patch is indented into the tendon with hundreds of fixation points into the tendon, significantly increasing the force-to-failure at the patch–tendon interface (154 N versus 221 N) [39]. In an ovine model, functional tissue ingrowth without excessive tissue reaction was shown. These results are very encouraging. However, it remains to support these findings in human studies.

#### 3.1.5. Nylon Patches

Currently, there is a lack of literature regarding the use of “nylon” patches, specifically Polyamide 6.6, in medical applications. Despite the widespread use of nylon in various industries, including textiles and engineering, its application in medical patches appears to be underexplored.

#### 3.1.6. Bioresorbable Scaffolds

Thon et al. conducted a recent study in twenty-three patients with large or massive full-thickness tears with a bio-inductive collagen patch applied on the bursal side during rotator cuff combined with a complete rotator cuff repair, noting its safety and demonstrating implant-induced tissue formation observed on MRI and ultrasound scans after 2 years with an imaging confirmed tendon healing rate of 96% but a 9% clinical failure [17].

Burkhard et al. also examined a bioabsorbable poly-4-hydroxybutyrate patch (Biofiber; Wright Medical, Memphis, TN, USA), finding that almost all repairs (14 out of 16) from October 2014 to January 2019 remained intact during the 1-year follow-up period after large posterosuperior rotator cuff tears (supraspinatus and/or infraspinatus), as evidenced by preoperative and postoperative MRI scans [40].

As shown in the prospective case study conducted by Chen et al. in 2021, involving 18 patients treated with either mini-open or arthroscopic surgical RC repair with the bio-active collagen scaffold (BCS), patients reported encouraging improvements in functional outcomes (ASES, OSS, and Constant-Murley scores), as well as quality of life assessments (AQoLs) and a reduction in VAS pain scores. MRI assessment at 12 months revealed complete healing in 64.8% of patients (11/17), three partial-thickness retears (17.6%), and three full-thickness retears (17.6%) [41].

In a subsequent randomized controlled study by Cai et al., 54 patients underwent rotator cuff repair with the suture-bridge technique and augmentation with 3D type I collagen patches (Zhejiang Xingyue Biotechnology, Hangzhou, China) to promote tendon-to-bone integration, whereas the control group was treated with the suture-bridge technique alone. Besides achieving significantly better clinical results measured with the Constant score, the results showed a retear rate of 13.7% in the study group compared with 34% in the control group, significantly reducing the retear rate. Tendon–bone integration was confirmed by biopsy specimens taken from the tendon–bone interface at 24 months postoperatively. Additionally, no adverse events were observed [42]. An important consideration in interpreting the findings of these studies is the limitations associated with small patient sample sizes.

### 3.2. PASTA Lesions

Indications and repair techniques for partial articular supraspinatus tendon avulsion (PASTA) have not yet reached a full consensus. The most widely used is the Ellman classification, with grade 1 as <33% of the tendon affected, grade 2 as 33–50% affected, and grade 3 as >50% affected [43]. A tear with >50% of the tendon thickness involved and resistant to conservative therapy can be eligible for repair, whilst tendons < 50% are nowadays mostly debrided. However, there is literature on suture repair for grade 2 lesions [44]. Different techniques exist for suturing, mostly a variation of a transtendinous suture or takedown and repair, all of them with advantages and complications [45,46]. One complication of these suture techniques is postoperative stiffness, as this is the most common complication after rotator cuff repair [47]. An alternative treatment proposed by Bushnell et al. is to augment the SSP with a patch by which the tension on the tendon is reduced, and the lesion can heal whilst incorporating the augmentation. In a prospective multicenter registry study of 272 patients with a mean age of 52.1 years, the tear size was assessed pre-operatively vs. post-operatively. Of the 272 tears, 49 were grade 1, 101 were grade 2, and 122 were grade 3 tears. A total of 241 patients were treated with isolated patch augmentation, whilst 31 had a takedown and repair + augmentation. Scores as ASES, SANE, WORC, VR-12 MCS, and PCS were measured for clinical relevance. Concomitant procedures included acromioplasty (94.9%), AC joint resection (46.3%), biceps tenodesis (41.9%), tenotomy (8.5%), labral repair (5.5%), capsular release (13.6%), and debridement not specified (59.9%). Patients with tears grade 2 or 3 with isolated augmentation had better scores at 2 and 6 weeks than those with takedown, repair, and augmentation. At 3 months, only the SANE and VR12 PCS differed, favoring the augmentation group, as at 1 year, no differences were found. A total of 11 revisions occurred, mostly for shoulder stiffness (five patients), but significant bursitis (three patients) of a dislodged graft (one patient) was reported [48]. The high number of concomitant interventions can influence the outcomes. Because there was no control group with repair alone, it is hard to draw conclusions from this technique, although we can confirm that it can be a possible valuable technique for treating these lesions. The data are still limited, and no RCT or meta-analysis exists on this topic, so a sensible amount of precaution is still required by using patch augmentations for this indication.

### 3.3. Biological Enhancement of Rotator Cuff Repairs

#### 3.3.1. Mesenchymal Stem Cells (MSCs)

Various methods were researched to improve the healing of tendons. MSCs are known to enhance tendon healing by speeding up matrix synthesis and adjusting the immune response [49]. Research has demonstrated that incorporating MSCs into surgical procedures can lower the rate of retears. In an article from Jiang et al., 3D-printed multilayer scaffolds with human adipose-derived MSCs (hADMSCs) embedded in a collagen-fibrin hydrogel showed promising results regarding mechanical as well as bioactivity [50].

#### 3.3.2. Platelet-Rich Plasma (PRP)

PRP contains various growth factors such as PDGF, TGF-B, FGF, IGF-1, IGF-2, IL-8, etc. Different forms are described, varying in the number of leukocytes and platelets, as well as the fibrin network [51]. Research indicates that platelet-rich therapies can positively influence tendon repair. Nonetheless, clinical evidence is mixed regarding whether this leads to enhanced tendon healing and better functional outcomes. A meta-analysis study conducted by Murley et al. proved that administering PRP during surgery significantly improved healing rates for small to medium and medium to large full-thickness tears [52].

#### 3.3.3. Vitamin D

Recent interest has focused on identifying factors that determine the success of rotator cuff repairs, with vitamin D levels emerging as a significant factor due to their link to bone and muscle proliferation and healing. Mechanisms by which vitamin D, particularly its activated form 1,25-dihydroxy-vitamin D, also known as calcitriol, influences osteoblast proliferation, bone mineral density, and skeletal muscle strength, all crucial for tendon-to-bone healing is discussed by Dougherty et al. [53] Despite limited studies, the promising role of vitamin D in tendon health suggests it could be beneficial in patch augmentation for rotator cuff injuries.

### 3.4. Complications

By implanting grafts, complications of this act arise. An increased immunological response, seen as graft rejection, was described by Adams et al. [19], but in recent studies, this complication does not arise anymore. Biological scaffolds such as Graft Jacket (Wright Medical Technology) patches are marketed as an acellular material, but researchers have detected DNA remnants on some of the commercially available grafts, possibly provoking inflammation reactions in rare cases. It is possible that due to processing methods remaining cell products are altered and no longer stimulate adverse events in host tissue or that the threshold values necessary for immunological reactions are not reached on this day [22].

Implanting foreign material comes with an increased risk of deep infection. Albers et al. described, in their study of 44 patients with a mean follow-up of 4.3 years with an open repair and augmented with a tissue-enhanced autologous rotator cuff repair [TEAR] patch, a deep infection rate of 7%, which is substantially higher than the reported infection rates of rotator cuff repair at 0.03–3.4%. In this same study, a failure of the patch was described in 11% of cases [54]. No other studies mention these complications. The graft integrity and incorporation were strongly correlated with the acromiohumeral distance; however, no correlation could be found with ROMs, PROMs, or patient satisfaction [55].

Non-degradable materials pose potential long-term risks due to foreign body reactions, which can hinder the final incorporation of tendons. This stands in contrast to degradable materials, for which no study has demonstrated any negative impact on tendon healing from remnants of degradation products [56,57].

Postoperative stiffness following partial tear repair is a widely recognized issue. Yeazell et al. conducted a study revealing significantly elevated stiffness rates within the bovine collagen patch group (Regeneten; Smith & Nephew, Memphis, TN, USA), with 8 out of 32 patients affected, compared with only 1 out of 32 in the control group. Additionally, 18% of patients in the patch group required reoperation to manage postoperative stiffness [58].

### 3.5. Cost Effectiveness

The economic burden of failed rotator cuff surgery is estimated at USD 200 million/year in the United States. The increase in cost of the implant and longer surgical times needs to be compared to the long-term benefits in principally reducing cost for rehabilitation, cuff revision, or arthroplasty surgery but also, and more difficult to estimate, the gain in productivity, i.e., return to work although this is a very difficult calculation which is also country dependent. Recent studies show a logical perioperative increase in costs by the use of implants, which, in most countries, is paid by government instances or medical insurance companies [59,60]. The estimated incremental cost-effectiveness ratio (ICER) (i.e., the additional cost to achieve one additional healed tendon for cuff augmentation vs. traditional repair for cuff augmentation) in the current literature is estimated at USD 13,061 and EUR 17,857 as calculated by McIntyre et al. in a US model and Rognoni et al. in an Italian model, respectively [61,62]. In the postoperative period, however, results show a decrease in costs for cuff augment surgery, which benefits society, mostly by reducing retear rates and thus revision cuff or arthroplasty surgery but also, for example, reducing work. The advantage increases as the tear enlarges or as the tissue quality reduces. Rognoni et al. suggest that the final social benefit is around EUR 5000 per healed tear [61]. Caution is required in analyzing these data as augmentations would reduce tear rates by 17.80%. Every company-specific product should be investigated independently; medical insurance or reimbursements are country-specific, and there is no consensus yet about the data on rotator cuff repair, let alone the data on augmentation.

## 4. Conclusions

The large heterogenicity in etiology, processing, mechanical aspects, and the potential to induce a biological stimulus of the rotator cuff augmentation products make it a difficult task to compare these entities and caution is required when analyzing data and drawing conclusions. It can be stated that for reinforcing a fully sutured large to massive rotator cuff, there can be an added value of reducing retear rates and increasing the thickness of the tendon, but when utilizing it as an interposition graft in partial repairs, the results are promising but still show high failure rates. The patch augmentation shows even promising results in treating PASTA lesions by only augmenting them. The additional complications, in general, seem to be limited, making it a safe procedure. Cost-effectiveness analyses indicate that while initial costs are higher, long-term savings from reduced rehabilitation, revision surgeries, and increased productivity can make patch augmentation economically beneficial. Large randomized control trials are still required in this field with special attention to tear size, repair pattern, and, last but not least, the patch specifics [63].

## Figures and Tables

**Table 1:** Summary of inclusion and exclusion criteria.

Inclusion Criteria	Exclusion Criteria
Randomized controlled trials (RCTs), cohort studies, case-control studies, case series, and systematic reviews/meta-analyses	Animal studies, in vitro studies
Patients diagnosed with rotator cuff tears who underwent surgical repair	Studies missing information about rotator cuff augmentation or techniques other than patch augmentation
Studies that assess outcomes such as graft incorporation, mechanical integrity, postoperative pain, range of motion, muscle strength, and retear rates	Studies that do not report on clinical or mechanical outcomes relevant to graft performance
Studies examining graft-related complications like infections, tissue rejection, and adverse events	Studies involving patients with comorbidities that significantly affect rotator cuff healing
